# Explicit Geodesic Projection Distance on the Statistical Manifold of Multivariate Elliptical Distributions

**DOI:** 10.3390/e28060701

**Published:** 2026-06-17

**Authors:** Xiangbing Chen, Yingying Wang, Jihong Xiao

**Affiliations:** 1 School of Mathematics and Statistics, Kashi University, Kashi 844000, China; wangyy260222@163.com; 2 Division of Mathematics, Sichuan University Jinjiang College, Meishan 620860, China; xiaojh2752@163.com

**Keywords:** information geometry, multivariate elliptical distribution, fisher metric, geodesic projection distance

## Abstract

Geodesic projection distances on statistical manifolds have been widely applied across various research fields. Nevertheless, the existing closed-form solution is only available for the Gaussian manifold. Multivariate elliptical distributions (MEDs) include the Gaussian, generalized Gaussian, Student’s *t* distribution, contaminated Gaussian, and other heavy-tailed models. Despite their widespread practical applicability, explicit geodesic projection solutions for MED manifolds remain unexplored. This paper derives the explicit geodesic projection distance from an arbitrary point on the MED manifold equipped with the Fisher metric onto its commonly used submanifold with a fixed mean vector. The core theoretical contribution lies in a novel symmetry-exploitation technique proposed to tackle the highly nonlinear and complex geodesic equations, and this methodological framework is readily extendable to other statistical manifolds. The derived results substantially advance the state-of-the-art by generalizing existing theories from the Gaussian manifold to the broader family of MEDs.

## 1. Introduction

Projection refers to finding the closest point in a subspace under a given metric or divergence and serves as a fundamental tool for solving various approximation problems. Information geometry treats the parameter space of probability density functions as a differentiable manifold, allowing intuitive interpretation and intrinsic analysis of systems involving stochastic uncertainty. As presented in [1], the manifold of exponential families admits a dually flat structure induced by Bregman divergence. The classical geodesic projection theorem yields a pair of dual projections, namely e-projection and m-projection, which provide optimal approximations on appropriate submanifolds in terms of Kullback–Leibler (KL) divergence. This framework has been successfully applied to integrated information theory [2,3,4], machine learning [5,6,7], matrix approximation [8,9,10], and other fields.

The Riemannian structure induced by the Fisher metric satisfies information monotonicity under one-to-one coordinate transformations and is invariant with respect to sufficient statistics reduction [1]. The corresponding geodesic distance, known as the Rao distance, provides a natural intrinsic measure of dissimilarity between probability distributions [11,12,13]. In information geometry, both the KL divergence and the Rao distance are widely adopted to capture high-order statistical information. Nevertheless, KL divergence is not a rigorous metric due to the lack of symmetry and the triangle inequality. In contrast, the Rao distance has been successfully applied to neural networks [14,15], signal processing [16,17], statistical inference [18,19], and other areas. Despite these advances, the closed-form Rao distance on the general Gaussian manifold remains elusive [20]. Recently, based on the explicit solution of geodesic equations on the Gaussian manifold with fixed initial point and direction [21], the geodesic projection distance has been explicitly derived in [22] and applied to distributed estimation fusion by minimizing the sum of squared geodesic distances to obtain the informative barycenter.

Multivariate elliptical distributions (MEDs), including multivariate Gaussian, generalized Gaussian, *t* distribution, contaminated Gaussian, and other heavy-tailed models, have been extensively used in practical modeling [23,24,25,26], owing to their robustness against outliers and modeling errors. Compared with the Gaussian manifold, the MED manifold endowed with the Fisher metric possesses considerably more complicated geodesic equations. Even the analytical expression of the Rao distance remains unknown for a given initial point and direction. Existing results focus on tight lower bounds (e.g., the Siegel distance [27]) and upper bounds (e.g., Manhattan-type distances [28]) for the Rao distance on the MED manifold.

Against this background, the main theoretical contributions of this paper are twofold:

(1) We derive the explicit closed-form expression for the geodesic projection distance from an arbitrary point on the MED manifold (equipped with the Fisher metric) onto its widely used submanifold with a fixed mean vector. To the best of our knowledge, this represents the first complete analytical solution to the geodesic projection problem for the general MED manifold, filling a long-standing gap in the information geometry of elliptical models.
(2) We develop a novel analytical technique that exploits the inherent symmetry embedded in the geodesic equations. This technique is not limited to the MED setting and can be extended to address geodesic projection problems on other statistical manifolds.

In terms of practical applicability, our results substantially generalize the existing geodesic projection theory from the Gaussian manifold to the broad class of MED manifolds, thereby expanding the scope of information-geometric methods to heavy-tailed and robust statistical modeling. Unlike the Gaussian-specific result in [22], the formula obtained in this paper applies to all MEDs with arbitrary generating functions, making them suitable for signal processing, distributed data fusion, and machine learning tasks where non-Gaussian and heavy-tailed observations are prevalent. By specializing our result to the Gaussian case, we recover the known geodesic projection distance in [22] as a direct corollary.

### Notations

Throughout this paper, $R^{m}$, $R^{m\times n}$, and $S_{+}^{m}$ denote the set of *m*-dimensional real column vectors, the set of m×n real matrices, and the set of m×m real symmetric positive-definite matrices, respectively. $I_{m}$ is the m×m identity matrix. The notation ∥·∥ represents the Euclidean norm for vectors and the Frobenius norm for matrices. For a square matrix A, $A^{\prime }$ and tr(A) stand for its transpose and trace, respectively. The expression A≻0 indicates that A is symmetric positive-definite. In addition, arccosh(·) denotes the inverse hyperbolic cosine function. $N_{m}\left(\mu ,\Sigma \right)$ represents an *m*-dimensional Gaussian distribution with mean μ and covariance matrix Σ. Finally, E[·] denotes the mathematical expectation operator.

## 2. Preliminaries

In this section, we briefly review fundamental concepts in information geometry and summarize key results that will be used throughout the paper.

### 2.1. Statistical Manifold and Fisher Metric

Consider a parametric statistical model

(1) $$\begin{array}{c} S=\left\{p\left(x;\theta \right):\theta =\left(\theta^{1},\ldots ,\theta^{m}\right)\in \Theta \right\} \end{array}$$

comprising probability density functions, where $\theta =(\theta^{1},\ldots ,\theta^{m})$ denotes the global coordinate system and Θ is an open subset of $R^{m}$. Endowed with the Levi–Civita connection ∇ and the Fisher metric *g* induced by the m×m Fisher information matrix G(θ), whose (i,j)-th entry is

(2) $$\begin{array}{c} g_{ij}\left(\theta \right)=E_{\theta }\left[\frac{\partial }{\partial \theta^{i}}\log p\left(x;\theta \right)\frac{\partial }{\partial \theta^{j}}\log p\left(x;\theta \right)\right], \end{array}$$

where $E_{\theta }\left[\cdot \right]$ denotes the expectation operator with respect to p(x;θ), the statistical manifold S admits a canonical Riemannian differentiable structure [1]. For notational simplicity, we identify a point p(x;θ)∈S with its coordinate vector θ.

Let $T_{\theta }S$ represent the tangent space at θ∈S. The Fisher metric defines an inner product on $T_{\theta }S$, denoted by

(3) $$\begin{array}{c} {\langle \cdot ,\cdot \rangle }_{\theta }:T_{\theta }S\times T_{\theta }S\to R. \end{array}$$

Accordingly, the length of a smooth curve segment γ(t) for $t\in [t_{0},t_{1}]$ is calculated via the integral

(4) $$\begin{array}{c} L\left(\gamma \right):=\int_{t_{0}}^{t_{1}}\sqrt{{\langle \dot{\gamma }\left(t\right),\dot{\gamma }\left(t\right)\rangle }_{\gamma (t)}}dt, \end{array}$$

where the superimposed dot denotes the derivative with respect to the parameter *t*.

### 2.2. Geodesic Distance and Variational Field

The geodesic distance (also known as the Rao distance) between two arbitrary points $\theta_{0},\theta_{1}\in S$ is obtained by solving the functional minimization problem

(5) $$\begin{array}{c} d\left(\theta_{0},\theta_{1}\right):=\min_{\gamma \in \Gamma }L\left(\gamma \right), \end{array}$$

where Γ denotes the set of all piecewise smooth curves connecting $\theta_{0}$ and $\theta_{1}$. On a complete Riemannian manifold S, the minimal length in (5) is achieved by a geodesic γ satisfying the zero-acceleration condition $\nabla_{\dot{\gamma }}\dot{\gamma }=0$. That is, the geodesic distance between two points coincides with the length of the geodesic segment joining them (see, e.g., [29]).

Let $\gamma :[t_{0},t_{1}]\to S$ be a piecewise differentiable curve on a Riemannian manifold S. A *variation* of γ is a differentiable mapping

(6) $$\begin{array}{c} \Phi :\left[t_{0},t_{1}\right]\times \left(-\epsilon ,\epsilon \right)\to S \end{array}$$

such that Φ(t,0)=γ(t). For each fixed u∈(−ϵ,ϵ), the curve $\Phi_{u}\left(t\right)=\Phi \left(t,u\right)$ is referred to as a varied curve of γ. Correspondingly, for a fixed *t*, the curve $\Phi_{t}\left(u\right)=\Phi \left(t,u\right)$ is called a transversal curve. The *variational field* of Φ is a piecewise differentiable vector field along γ(t), defined as the velocity vector of the transversal curve at u=0, i.e.,

(7) $$\begin{array}{c} V\left(t\right)=\frac{\partial \Phi_{t}}{\partial u}{|}_{u=0}. \end{array}$$

Let $\rho \left(u\right)=\rho \left(\Phi_{u}\right)$ denote the length of the varied curve $\Phi_{u}\left(t\right)$. The first variation formula of arc length (see, e.g., [29]) is given by

(8) $$\begin{array}{c} \frac{d\rho (u)}{du}{|}_{u=0}=\frac{t_{1}-t_{0}}{\rho }\left(\langle \dot{\gamma },V\rangle {|}_{t_{0}}^{t_{1}}-\int_{t_{0}}^{t_{1}}\langle V,\nabla_{\dot{\gamma }}\dot{\gamma }\rangle dt\right), \end{array}$$

where ρ=ρ(0) represents the length of the original curve γ.

### 2.3. Information Geometry of Multivariate Elliptical Distributions

An *m*-dimensional random vector x follows a multivariate elliptical distribution (MED), denoted by ${\mathrm{EL}}_{m}^{h}\left(\mu ,\Sigma \right)$, if its probability density function (pdf) takes the form

(9) $$p_{h}\left(x;\mu ,\Sigma \right)={\left|\Sigma \right|}^{-1/2}h\left({\left(x-\mu \right)}^{\prime }\Sigma^{-1}\left(x-\mu \right)\right)$$

for some generating function h(·), where μ is the mean vector and Σ≻0 is the scatter matrix. Representative examples include the multivariate generalized Gaussian distribution (MGGD) with shape parameter β and the multivariate *t* distribution (MTD) with ν degrees of freedom, whose generating functions are respectively given by

(10) $$\begin{array}{c} h\left(u\right)=\frac{\beta \Gamma (m/2)}{\pi^{m/2}\Gamma \left(m/\left(2\beta \right)\right)2^{m/(2\beta )}}\exp\left(-\frac{1}{2}u^{\beta }\right) \end{array}$$

and

(11) $$\begin{array}{c} h\left(u\right)=\frac{\Gamma ((\nu +m)/2)}{{\left(\pi \nu \right)}^{m/2}\Gamma \left(\nu /2\right)}{\left(1+\frac{u}{\nu }\right)}^{-(\nu +m)/2}. \end{array}$$

Let Ω be the lower-triangular Cholesky factor of Σ, $z=\Omega^{-1}\left(x-\mu \right)$, and $\xi ={d\log(h(∥z∥}^{2}))/{d(∥z∥}^{2})$. Define two constants

(12) $$\begin{array}{c} a_{h}=\frac{1}{m}E\left[\xi^{2}{∥z∥}^{2}\right],\;b_{h}=\frac{1}{m(m+2)}E\left[\xi^{2}{∥z∥}^{4}\right]. \end{array}$$

Substituting (10) and (11) into (12) yields

(13) $$\begin{array}{c} a_{h}=\frac{\beta^{2}\Gamma \left(2+\frac{m}{2\beta }-\frac{1}{\beta }\right)}{2^{\frac{1}{\beta }}m\Gamma \left(\frac{m}{2\beta }\right)},\;b_{h}=\frac{m+2\beta }{4m+8} \end{array}$$

for the MGGD, and

(14) $$\begin{array}{c} a_{h}=b_{h}=\frac{\nu +m}{4(\nu +m+2)} \end{array}$$

for the MTD.

Equipped with the Fisher metric and Levi–Civita connection, the statistical model

(15) $$\begin{array}{c} M=\{{\mathrm{EL}}_{m}^{h}\left(\mu ,\Sigma \right):\mu \in R^{m},\;\Sigma \in S_{+}^{m}\} \end{array}$$

with a fixed generating function *h* and global coordinates θ=(μ,Σ) forms a Riemannian manifold. According to [27,30], the squared line element at a point θ=(μ,Σ) is

(16) $$\begin{array}{c} ds^{2}=4a_{h}d\mu^{\prime }\Sigma^{-1}d\mu +2b_{h}\mathrm{tr}{\left(\Sigma^{-1}d\Sigma \right)}^{2}+\frac{4b_{h}-1}{4}{\left[\mathrm{tr}\left(\Sigma^{-1}d\Sigma \right)\right]}^{2}, \end{array}$$

and the geodesic equations on M are given by

(17) $$\begin{array}{cc} \; & {\ddot{\mu }}_{t}-{\dot{\Sigma }}_{t}\Sigma_{t}^{-1}{\dot{\mu }}_{t}=0, \end{array}$$

(18) $$\begin{array}{cc} \; & {\ddot{\Sigma }}_{t}+a{\dot{\mu }}_{t}{\dot{\mu }}_{t}^{\prime }-b{\dot{\mu }}_{t}^{\prime }\Sigma_{t}^{-1}{\dot{\mu }}_{t}\Sigma_{t}-{\dot{\Sigma }}_{t}\Sigma_{t}^{-1}{\dot{\Sigma }}_{t}=0, \end{array}$$

where

(19) $$\begin{array}{c} a=\frac{a_{h}}{b_{h}},\;b=\frac{a_{h}\left(4b_{h}-1\right)}{8b_{h}^{2}+mb_{h}\left(4b_{h}-1\right)}. \end{array}$$

Here, the subscript *t* attached to a variable signifies that the variable depends on *t*, while the superimposed dot represents differentiation with respect to the arc-length parameter *t*.

To date, the derivation of closed-form expressions for the Rao distance and geodesic curves on the general MED manifold M with m>1 still remains an open problem. Explicit closed-form solutions are only available for several special submanifolds subject to a fixed mean vector or fixed scatter matrix (see, e.g., [31]).

## 3. Existence of Geodesic Projection onto Fixed-Mean Submanifold

**Proposition** **1.** *On the MED manifold M, we have the following results:*

(20) $$\begin{array}{c} a>0,\;a-b>0. \end{array}$$

**Proof.**  It is evident from (12) that $a_{h}>0$ and $b_{h}>0$. Moreover, by the Cauchy–Schwartz inequality, for any $A\in R^{m\times m}$,

(21) $$\begin{array}{c} \mathrm{tr}\left(A^{2}\right)\geq \frac{1}{m}{\mathrm{tr}}^{2}\left(A\right) \end{array}$$

with equality if and only if $A=cI_{m}$ for any c∈R. Substituting dμ=0 into (16) then yields

(22) $$\begin{array}{cc} ds^{2} & =2b_{h}\mathrm{tr}{\left(\Sigma^{-1}d\Sigma \right)}^{2}+\frac{4b_{h}-1}{4}{\mathrm{tr}}^{2}\left(\Sigma^{-1}d\Sigma \right)\geq \left(\frac{2b_{h}}{m}+\frac{4b_{h}-1}{4}\right){\mathrm{tr}}^{2}\left(\Sigma^{-1}d\Sigma \right). \end{array}$$

Hence, for any **Σ** and dΣ, the above inequality implies $2b_{h}/m+\left(4b_{h}-1\right)/4>0$, or equivalently,

(23) $$\begin{array}{c} b_{h}>\frac{m}{4m+8}, \end{array}$$

Combined with (19), we readily obtain a>0 and a−b>0. □

Define the geodesic projection distance from a given point $\theta_{0}=\left(\mu_{0},\Sigma_{0}\right)$ in M onto its submanifold

(24) $$\begin{array}{c} M_{\mu }=\left\{{\mathrm{EL}}_{m}^{h}\left(\mu ,\Sigma \right):\Sigma \in S_{+}^{m}\right\} \end{array}$$

with a fixed mean vector $\mu \in R^{m}$ by

(25) $$\begin{array}{c} \rho :=d\left(\theta_{0},M_{\mu }\right)=\underset{\theta \in M_{\mu }}{\inf}d\left(\theta_{0},\theta \right). \end{array}$$

This distance is well defined by the theorem presented below.

**Theorem** **1.** *The following statements hold:*

*(1)*  *The MED manifold M is a complete Riemannian manifold and $M_{\mu }$ is a closed submanifold of M.*
*(2)*  *For any $\theta_{0}\in M$ and $\theta_{0}\notin M_{\mu }$, there exists a point $\theta_{P}\in M_{\mu }$ such that the geodesic segment connecting $\theta_{0}$ and $\theta_{P}$ is orthogonal to the submanifold $M_{\mu }$, i.e., $\dot{\Sigma }\left(\rho \right)=0$, and its length realizes the minimum of $d(\theta_{0},\theta )$ over all $\theta \in M_{\mu }$.*

**Proof.** 

(1) The manifold M of multivariate elliptical distributions ${\mathrm{EL}}_{m}^{h}\left(\mu ,\Sigma \right)$ is topologically isomorphic to
  (26) $$\begin{array}{c} M≅R^{m}\times S_{+}^{m}. \end{array}$$
  From (16), the Fisher metric on M decomposes into a product metric
  (27) $$\begin{array}{c} g=g_{\mu }⊕g_{\Sigma }. \end{array}$$
  Here $g_{\mu }=4a_{h}d\mu^{\prime }\Sigma^{-1}d\mu$ is a Euclidean-type metric, and thus the Riemannian manifold $\left(R^{m},g_{\mu }\right)$ is complete. Meanwhile, $g_{\Sigma }=2b_{h}\mathrm{tr}{\left(\Sigma^{-1}d\Sigma \right)}^{2}+\frac{4b_{h}-1}{4}{\left[\mathrm{tr}\left(\Sigma^{-1}d\Sigma \right)\right]}^{2}$ is an affine-invariant metric defined on the symmetric positive definite cone $S_{+}^{m}$, which is known to be complete (see, e.g., [27]). Since the product of two complete Riemannian manifolds is complete, M is both geodesically and metrically complete. By the Hopf–Rinow theorem (see, e.g., [29]), M is therefore a complete Riemannian manifold. Additionally, since the projection from M onto $R^{m}$ is continuous, its preimage $M_{\mu }$ is closed in M.
(2) By the continuity of the distance function d(·,·) and the completeness of M, the Hopf–Rinow theorem ensures the existence of a point $\theta_{P}\in M_{\mu }$ such that $\rho =d\left(\theta_{0},\theta_{P}\right)=d\left(\theta_{0},M_{\mu }\right)$. Consider the geodesic segment $\gamma \left(t\right)=\theta \left(t\right)=\left(\mu_{t},\Sigma_{t}\right)$ with arc-length parameter t∈[0,ρ], connecting $\theta_{0}$ to its geodesic projection $\theta_{\rho }=\theta_{P}=\left(\mu ,\Sigma \left(\rho \right)\right)$ in $M_{\mu }$.
  Now let $v\in T_{\theta_{P}}M_{\mu }$ be arbitrary. There exists a curve β(u),u∈(−ϵ,ϵ) in M, satisfying the initial conditions $\beta \left(0\right)=\theta_{P}$ and $\dot{\beta }\left(0\right)=v$. For each u∈(−ϵ,ϵ), set $\rho \left(u\right):=\rho \left(\gamma_{u}\right)=d\left(\theta_{0},\beta \left(u\right)\right)$, and let $\gamma_{u}\left(t\right),t\in \left[0,\rho \left(u\right)\right]$ be a geodesic from $\theta_{0}$ to β(u). Then $\Phi \left(t,u\right):=\gamma_{u}\left(t\right)$ yields a variation in the geodesic $\gamma_{0}\left(t\right)=\gamma \left(t\right)$. Applying the first variation formula of arc length (8), we obtain
  (28) $$\begin{array}{cc} \frac{d\rho (u)}{du}{|}_{u=0} & =\langle \dot{\gamma }\left(t\right),V\left(t\right)\rangle {|}_{0}^{\rho }-\int_{0}^{\rho }\langle V\left(t\right),\nabla_{\dot{\gamma }}\dot{\gamma }\rangle dt\\ \; & =\langle \dot{\gamma }\left(t\right),V\left(t\right)\rangle {|}_{0}^{\rho }\\ \; & =\langle \dot{\gamma }\left(\rho \right),v\rangle \\ \; & =0, \end{array}$$
  where ρ=ρ(0) is the length of γ, and V is the variational field with V(0)=0 and V(ρ)=v. As $\langle \dot{\gamma }\left(\rho \right),v\rangle =0$ holds for all v, we conclude that $\dot{\gamma }\left(\rho \right)=0$. This implies that the **Σ**-component of the tangent vector of γ at $\theta_{P}$ is given by
  (29) $$\dot{\Sigma }\left(\rho \right)=0.$$

□

By Theorem 1, there exists a point in $M_{\mu }$ at which the infimum distance $\rho =d(\theta_{0},M_{\mu })$ defined in (25) is attained.

## 4. Explicit Geodesic Projection Distance onto Fixed-Mean Submanifold

To further deduce the explicit geodesic distance, the following theorem gives the first-order form of the geodesic Equations (17) and (18) on M.

**Theorem** **2.** *The geodesic Equations* (17)* and *(18)* can be reduced to the first order form as*

(30) $$\begin{array}{cc} {\dot{\mu }}_{t} & =\Sigma_{t}v_{0}, \end{array}$$

(31) $$\begin{array}{cc} {\dot{\Sigma }}_{t} & =\Sigma_{t}\left(B_{0}-av_{0}\mu_{t}^{\prime }+b\mu_{t}^{\prime }v_{0}I_{m}\right), \end{array}$$

*where $v_{0}\in R^{m}$ and $B_{0}\in R^{m\times m}$ are an arbitrary constant vector and integration matrix, respectively.*

**Proof.**  Left-multiplying both sides of the first geodesic Equation (17) by $\Sigma_{t}^{-1}$ yields

(32) $$\begin{array}{c} \Sigma_{t}^{-1}{\ddot{\mu }}_{t}-\Sigma_{t}^{-1}{\dot{\Sigma }}_{t}\Sigma_{t}^{-1}{\dot{\mu }}_{t}=0, \end{array}$$

and then

(33) $$\begin{array}{c} d(\Sigma_{t}^{-1}{\dot{\mu }}_{t})=0, \end{array}$$

from which it follows that

(34) $$\begin{array}{c} {\dot{\mu }}_{t}=\Sigma_{t}v_{0} \end{array}$$

holds for any $v_{0}\in R^{m}$.

Further left-multiplying both sides of the second geodesic Equation (18) by $\Sigma_{t}^{-1}$ gives

(35) $$\begin{array}{c} \Sigma_{t}^{-1}{\ddot{\Sigma }}_{t}-{\left(\Sigma_{t}^{-1}{\dot{\Sigma }}_{t}\right)}^{2}+a\Sigma_{t}^{-1}{\dot{\mu }}_{t}{\dot{\mu }}_{t}^{\prime }-b{\dot{\mu }}_{t}^{\prime }\Sigma_{t}^{-1}{\dot{\mu }}_{t}I_{m}=0. \end{array}$$

Substituting (34) into the above expression leads to

(36) $$\begin{array}{c} \Sigma_{t}^{-1}{\ddot{\Sigma }}_{t}-\Sigma_{t}^{-1}{\dot{\Sigma }}_{t}\Sigma_{t}^{-1}{\dot{\Sigma }}_{t}+av_{0}{\dot{\mu }}_{t}^{\prime }-b{\dot{\mu }}_{t}^{\prime }v_{0}I_{m}=0. \end{array}$$

It is straightforward to verify that

(37) $$\begin{array}{c} d\left(\Sigma_{t}^{-1}{\dot{\Sigma }}_{t}\right)+av_{0}d\left(\mu_{t}^{\prime }\right)-bd\left(\mu_{t}^{\prime }\right)v_{0}I_{m}=0, \end{array}$$

which implies the existence of an arbitrary matrix $B_{0}\in R^{m\times m}$ satisfying

(38) $$\begin{array}{c} \Sigma_{t}^{-1}{\dot{\Sigma }}_{t}+av_{0}\mu_{t}^{\prime }-b\mu_{t}^{\prime }v_{0}I_{m}=B_{0}. \end{array}$$

Finally, rearranging the terms of the above equation directly yields (31). □

From the squared line element (16), the norm of the tangent vector along the geodesic satisfies the normalization condition

(39) $$\begin{array}{c} 4a_{h}{\dot{\mu }}_{t}^{\prime }\Sigma_{t}^{-1}{\dot{\mu }}_{t}+2b_{h}\mathrm{tr}{\left(\Sigma_{t}^{-1}{\dot{\Sigma }}_{t}\right)}^{2}+\frac{4b_{h}-1}{4}{\mathrm{tr}}^{2}\left(\Sigma_{t}^{-1}{\dot{\Sigma }}_{t}\right)=1. \end{array}$$

Substituting Equations (29) and (30) into (39) then yields

(40) $$\begin{array}{c} 4a_{h}v_{0}^{\prime }\Sigma_{\rho }v_{0}=1. \end{array}$$

We proceed to derive several key results in Propositions 2–4 concerning the explicit form of the geodesic projection distance $\rho =d(\theta_{0},M_{\mu })$ from $\theta_{0}=\left(\mu_{0},\Sigma_{0}\right)\in M$ onto the submanifold $M_{\mu }$ of M. As the geodesic projection $\theta_{\rho }=\left(\mu_{\rho },\Sigma_{\rho }\right)$ belongs to the fixed-mean submanifold $M_{\mu }$, it follows that $\mu_{\rho }=\mu$, where $\mu_{\rho }$ stands for $\mu_{t}$ evaluated at t=ρ with no ambiguity incurred.

**Proposition** **2.** *The geodesic projection curve $\theta_{t}=\left(\mu_{t},\Sigma_{t}\right)$ connecting $\theta_{0}$, and the geodesic projection $\theta_{\rho }\in M_{\mu }$ satisfies*

(41) $$\begin{array}{c} v_{0}^{\prime }\left(\Sigma_{t}-\Sigma_{0}\right)=\frac{a-b}{2}v_{0}^{\prime }\left(\left(\mu_{\rho }-\mu_{0}\right){\left(\mu_{\rho }-\mu_{0}\right)}^{\prime }-\left(\mu_{\rho }-\mu_{t}\right){\left(\mu_{\rho }-\mu_{t}\right)}^{\prime }\right), \end{array}$$

*where $\mu_{\rho }=\mu$ is a constant vector.*

**Proof.**  We first compute several matrix-valued integrals that will be used in the sequel. Let $\mu_{t}={\left[\mu_{t1},\ldots ,\mu_{tm}\right]}^{\prime }$ along the geodesic segment $\theta_{t}=\left(\mu_{t},\Sigma_{t}\right),\;t\in \left[0,\rho \right]$. Integration by parts readily gives

(42) $$\begin{array}{c} \int_{0}^{t}\left(\mu_{\rho i}-\mu_{si}\right){\dot{\mu }}_{sj}ds=\left(\mu_{\rho i}-\mu_{ti}\right)\mu_{tj}-\left(\mu_{\rho i}-\mu_{0i}\right)\mu_{0j}+\int_{0}^{t}\mu_{sj}d\mu_{si} \end{array}$$

and

(43) $$\begin{array}{c} \int_{0}^{t}\left(\mu_{\rho j}-\mu_{sj}\right){\dot{\mu }}_{si}ds=\left(\mu_{ti}-\mu_{0i}\right)\mu_{\rho j}-\int_{0}^{t}\mu_{sj}d\mu_{si}. \end{array}$$

From Equation (31) together with the tangential condition ${\dot{\Sigma }}_{\rho }=\dot{\Sigma }\left(\rho \right)=0$ at the projection $\theta_{\rho }$, we obtain

(44) $$\begin{array}{c} B_{0}-av_{0}\mu_{\rho }^{\prime }+b\mu_{\rho }^{\prime }v_{0}I_{m}=0, \end{array}$$

which, when combined with (30), (31) and (44), implies

(45) $$\begin{array}{c} {\dot{\Sigma }}_{t}=a{\dot{\mu }}_{t}{\left(\mu_{\rho }-\mu_{t}\right)}^{\prime }-b{\left(\mu_{\rho }-\mu_{t}\right)}^{\prime }v_{0}\Sigma_{t}. \end{array}$$

It is clear from (45) that ${\dot{\mu }}_{t}{\left(\mu_{\rho }-\mu_{t}\right)}^{\prime }$ is symmetric, i.e.,

(46) $$\begin{array}{c} {\dot{\mu }}_{ti}\left(\mu_{\rho j}-\mu_{tj}\right)={\dot{\mu }}_{tj}\left(\mu_{\rho i}-\mu_{ti}\right) \end{array}$$

for i,j=1,…,m.

Furthermore, using (42), (43) and (46), we derive

(47) $$\begin{array}{c} \int_{0}^{t}\mu_{sj}d\mu_{si}=\frac{1}{2}\left(\mu_{\rho j}\mu_{ti}-\mu_{\rho j}\mu_{0i}+\mu_{\rho i}\mu_{0j}-\mu_{0i}\mu_{0j}-\mu_{\rho i}\mu_{tj}+\mu_{ti}\mu_{tj}\right), \end{array}$$

and then

(48) $$\begin{array}{c} \int_{0}^{t}\left(\mu_{\rho j}-\mu_{sj}\right){\dot{\mu }}_{si}ds=\frac{1}{2}\left(\mu_{\rho i}-\mu_{0i}\right)\left(\mu_{\rho j}-\mu_{0j}\right)-\frac{1}{2}\left(\mu_{\rho i}-\mu_{ti}\right)\left(\mu_{\rho j}-\mu_{tj}\right), \end{array}$$

so that

(49) $$\begin{array}{c} \int_{0}^{t}{\dot{\mu }}_{s}{\left(\mu_{\rho }-\mu_{s}\right)}^{\prime }ds=\frac{1}{2}\left(\mu_{\rho }-\mu_{0}\right){\left(\mu_{\rho }-\mu_{0}\right)}^{\prime }-\frac{1}{2}\left(\mu_{\rho }-\mu_{t}\right){\left(\mu_{\rho }-\mu_{t}\right)}^{\prime }. \end{array}$$

Substituting (30) and

(50) $$\begin{array}{c} {\dot{\mu }}_{t}^{\prime }{\left(\mu_{\rho }-\mu_{t}\right)}^{\prime }v_{0}=v_{0}^{\prime }\left(\mu_{\rho }-\mu_{t}\right){\dot{\mu }}_{t}^{\prime }=v_{0}^{\prime }{\dot{\mu }}_{t}{\left(\mu_{\rho }-\mu_{t}\right)}^{\prime } \end{array}$$

due to the symmetry of ${\dot{\mu }}_{t}{\left(\mu_{\rho }-\mu_{t}\right)}^{\prime }$ into (45) yields

(51) $$\begin{array}{cc} v_{0}^{\prime }{\dot{\Sigma }}_{t} & =av_{0}^{\prime }{\dot{\mu }}_{t}{\left(\mu_{\rho }-\mu_{t}\right)}^{\prime }-bv_{0}^{\prime }\Sigma_{t}{\left(\mu_{\rho }-\mu_{t}\right)}^{\prime }v_{0}=\left(a-b\right)v_{0}^{\prime }{\dot{\mu }}_{t}{\left(\mu_{\rho }-\mu_{t}\right)}^{\prime }. \end{array}$$

Finally, integrating both hand sides of Equation (51) over [0,t] by the matrix-valued integral (49), we obtain

(52) $$\begin{array}{c} v_{0}^{\prime }\left(\Sigma_{t}-\Sigma_{0}\right)=\frac{a-b}{2}v_{0}^{\prime }\left(\left(\mu_{\rho }-\mu_{0}\right){\left(\mu_{\rho }-\mu_{0}\right)}^{\prime }-\left(\mu_{\rho }-\mu_{t}\right){\left(\mu_{\rho }-\mu_{t}\right)}^{\prime }\right). \end{array}$$

□

**Proposition** **3.** *There exists a scalar function $\lambda_{t}$ for each t∈[0,ρ] such that*

(53) $$\begin{array}{c} {\dot{\mu }}_{t}=\Sigma_{t}v_{0}=\lambda_{t}\left(\mu_{\rho }-\mu_{t}\right). \end{array}$$

**Proof.**  It is readily seen from (45) that ${\dot{\mu }}_{t}{\left(\mu_{\rho }-\mu_{t}\right)}^{\prime }$ is symmetric. Together with (30), this implies that $\Sigma_{t}v_{0}{\left(\mu_{\rho }-\mu_{t}\right)}^{\prime }$ is also symmetric. Hence, there exists a scalar function $\lambda_{t}$ for any t∈[0,ρ] satisfying (53). □

**Proposition** **4.** *For any t∈[0,ρ],*

(54) $$\begin{array}{c} v_{0}^{\prime }\left(\mu_{t}-\mu_{\rho }\right)=\alpha \frac{e^{\beta (t-\rho )}-1}{e^{\beta (t-\rho )}+1}, \end{array}$$

*where*

(55) $$\begin{array}{c} \alpha =\frac{1}{\sqrt{2a_{h}\left(a-b\right)}},\;\beta =\sqrt{\frac{a-b}{2a_{h}}}. \end{array}$$

**Proof.**  From (41), we immediately obtain

(56) $$\begin{array}{c} v_{0}^{\prime }\Sigma_{t}=v_{0}^{\prime }\Sigma_{\rho }-\frac{a-b}{2}v_{0}^{\prime }\left(\mu_{\rho }-\mu_{t}\right){\left(\mu_{\rho }-\mu_{t}\right)}^{\prime }. \end{array}$$

Left-multiplying both sides of the geodesic Equation (30) by $v_{0}^{\prime }$ and inserting (56) yields

(57) $$\begin{array}{c} v_{0}^{\prime }{\dot{\mu }}_{t}=v_{0}^{\prime }\Sigma_{\rho }v_{0}-\frac{a-b}{2}{\left(v_{0}^{\prime }\left(\mu_{t}-\mu_{\rho }\right)\right)}^{2}. \end{array}$$

Define

(58) $$x_{t}:=v_{0}^{\prime }\left(\mu_{t}-\mu_{\rho }\right)$$

and

(59) $$C_{0}:=v_{0}^{\prime }\Sigma_{\rho }v_{0}=\frac{1}{4a_{h}},$$

where the second equality in (59) follows from the normalizing condition (40). Then (57) implies

(60) $$\begin{array}{c} {\dot{x}}_{t}=C_{0}-\frac{a-b}{2}x_{t}^{2}. \end{array}$$

Separating variables and integrating both sides of (60) give

(61) $$\begin{array}{c} \frac{\alpha +x_{t}}{\alpha -x_{t}}=Ce^{(a-b)\alpha t}, \end{array}$$

where $\alpha =\sqrt{2C_{0}/\left(a-b\right)}$ and *C* is an arbitrary real constant. From the boundary condition $\mu \left(\rho \right)=\mu_{\rho }$, we have $x_{\rho }=0$, which in turn gives $C=e^{-(a-b)\alpha \rho }$. We thus derive

(62) $$\begin{array}{c} x_{t}=\alpha \frac{e^{\beta (t-\rho )}-1}{e^{\beta (t-\rho )}+1} \end{array}$$

with β=(a−b)α, from which (54) and (55) follow immediately. □

Having finalized all preliminary developments, we are ready to establish the explicit formula for the geodesic projection distance.

**Theorem** **3.** *The geodesic projection distance from $\theta_{0}=\left(\mu_{0},\Sigma_{0}\right)$ in M onto $M_{\mu }$ is given by*

(63) $$\begin{array}{c} \rho =\frac{1}{\beta }\mathrm{arccosh}\left(1+\left(a-b\right){\left(\mu_{\rho }-\mu_{0}\right)}^{\prime }\Sigma_{0}^{-1}\left(\mu_{\rho }-\mu_{0}\right)\right) \end{array}$$

*with $\beta =\sqrt{\frac{a-b}{2a_{h}}}$ and $\mu_{\rho }=\mu$.*

**Proof.**  Setting t=0 and left-multiplying both sides of (53) by $\Sigma_{0}^{-1}$, we obtain

(64) $$\lambda_{0}\Sigma_{0}^{-1}\left(\mu_{\rho }-\mu_{0}\right)=v_{0},$$

which further implies

(65) $$\lambda_{0}^{2}\Sigma_{0}^{-1}\left(\mu_{\rho }-\mu_{0}\right){\left(\mu_{\rho }-\mu_{0}\right)}^{\prime }\Sigma_{0}^{-1}\Sigma_{\rho }=v_{0}v_{0}^{\prime }\Sigma_{\rho }.$$

Moreover, taking the trace operation on both sides of (65) and applying the normalizing condition (40) yield

(66) $$\begin{array}{c} \lambda_{0}^{2}\mathrm{tr}\left(\Sigma_{0}^{-1}\left(\mu_{\rho }-\mu_{0}\right){\left(\mu_{\rho }-\mu_{0}\right)}^{\prime }\Sigma_{0}^{-1}\Sigma_{\rho }\right)=\frac{1}{4a_{h}}. \end{array}$$

From (40) and (56), it is straightforward to see that

(67) $$\begin{array}{c} v_{0}^{\prime }\left(\Sigma_{t}+\frac{a-b}{2}\left(\mu_{\rho }-\mu_{t}\right){\left(\mu_{\rho }-\mu_{t}\right)}^{\prime }\right)v_{0}=\frac{1}{4a_{h}}, \end{array}$$

and substituting (54) and (55) into the above leads to

(68) $$\begin{array}{cc} v_{0}^{\prime }\Sigma_{t}v_{0} & =\frac{1}{4a_{h}}-\frac{a-b}{2}v_{0}^{\prime }\left(\mu_{\rho }-\mu_{t}\right){\left(\mu_{\rho }-\mu_{t}\right)}^{\prime }v_{0}\\ \; & =\frac{1}{a_{h}}\frac{e^{\beta (t-\rho )}}{{\left(e^{\beta (t-\rho )}+1\right)}^{2}}. \end{array}$$

On the other hand, left-multiplying both sides of (53) by $v_{0}^{\prime }$ and inserting (54) and (68) allow us to derive the closed-form expression for the scalar function $\lambda_{t}$ as

(69) $$\lambda_{t}=\frac{1}{a_{h}\alpha }\frac{e^{\beta (t-\rho )}}{1-e^{2\beta (t-\rho )}}.$$

In addition, from (53) we have $v_{0}=\lambda_{0}\Sigma_{0}^{-1}\left(\mu_{\rho }-\mu_{0}\right)$. Substituting this into (41) gives

(70) $$\begin{array}{c} {\left(\mu_{\rho }-\mu_{0}\right)}^{\prime }\Sigma_{0}^{-1}\Sigma_{\rho }={\left(\mu_{\rho }-\mu_{0}\right)}^{\prime }+\frac{a-b}{2}d_{M}{\left(\mu_{\rho }-\mu_{0}\right)}^{\prime }, \end{array}$$

where $d_{M}={\left(\mu_{\rho }-\mu_{0}\right)}^{\prime }\Sigma_{0}^{-1}\left(\mu_{\rho }-\mu_{0}\right)$. Right-multiplying both sides of (70) by $\Sigma_{0}^{-1}\left(\mu_{\rho }-\mu_{0}\right)$ and taking the trace then yields

(71) $$\begin{array}{c} \mathrm{tr}\left(\Sigma_{0}^{-1}\left(\mu_{\rho }-\mu_{0}\right){\left(\mu_{\rho }-\mu_{0}\right)}^{\prime }\Sigma_{0}^{-1}\Sigma_{\rho }\right)=d_{M}+\frac{a-b}{2}d_{M}^{2}. \end{array}$$

Combining (71) and (66) with (69), we conclude that

(72) $$\begin{array}{c} d_{M}+\frac{a-b}{2}d_{M}^{2}=\frac{1}{8(a-b)}{\left(e^{\beta \rho }-e^{-\beta \rho }\right)}^{2}. \end{array}$$

Further computation then leads to the explicit geodesic distance

(73) $$\begin{array}{c} \rho =\frac{1}{\beta }\mathrm{arccosh}\left(1+\left(a-b\right)d_{M}\right). \end{array}$$

 □

When restricted to the Gaussian distribution, the geodesic projection distance derived in this paper reduces to the classical result presented in [22].

**Corollary** **1.** *For a point $\theta_{0}=\left(\mu_{0},\Sigma_{0}\right)$ in the Gaussian manifold*

(74) $$\begin{array}{c} N=\left\{N_{m}\left(\mu ,\Sigma \right):\mu \in R^{m},\;\Sigma \in S_{+}^{m}\right\}, \end{array}$$

*and its submanifold with a fixed mean vector μ*

(75) $$\begin{array}{c} N_{\mu }=\left\{N_{m}\left(\mu ,\Sigma \right):\Sigma \in S_{+}^{m}\right\}, \end{array}$$

*the geodesic projection distance is given by*

(76) $$\begin{array}{c} \rho =\frac{1}{\sqrt{2}}\mathrm{arccosh}\left(1+{\left(\mu -\mu_{0}\right)}^{\prime }\Sigma_{0}^{-1}\left(\mu -\mu_{0}\right)\right). \end{array}$$

**Proof.**  For the Gaussian distribution, the parameters can be directly calculated as

(77) $$\begin{array}{c} a_{h}=\frac{1}{4},\;b_{h}=\frac{1}{4}, \end{array}$$

which further leads to

(78) $$\begin{array}{c} a=1,\;b=0,\;\beta =\sqrt{2} \end{array}$$

through (19) and (55). Substituting these parameters into the geodesic projection distance formula (63) established in this paper yields

(79) $$\begin{array}{c} \rho =\frac{1}{\sqrt{2}}\mathrm{arccosh}\left(1+{\left(\mu -\mu_{0}\right)}^{\prime }\Sigma_{0}^{-1}\left(\mu -\mu_{0}\right)\right), \end{array}$$

which coincides with the result in [22]. □

## 5. Conclusions

In this paper, we derive an explicit closed-form expression for the geodesic projection distance from an arbitrary point on the MED manifold with Fisher metric to its widely used submanifold with a fixed mean vector. A new analytical approach is developed to solve complex geodesic equations by fully exploiting the inherent symmetric structure, providing a new tool for tackling geodesic projection problems on general statistical manifolds. Our results significantly extend the existing geodesic projection theory from the Gaussian manifold to a broader class of multivariate elliptical distributions, including Gaussian, generalized Gaussian, Student’s *t* distribution, contaminated Gaussian, and other heavy-tailed models. Specializing the derived formula to the Gaussian case directly recovers the classical result as a corollary, which confirms the validity and unifying nature of the proposed framework. Future research directions include deriving the explicit geodesic projection onto the MED submanifold with a fixed mean vector, extending the proposed approach to more general statistical manifolds, and investigating its potential applications in related fields.

## Data Availability

This work is a purely theoretical investigation without experimental, simulated or observational datasets. No new data were generated or analyzed throughout the research, so data sharing is not applicable to this article.
